# Transaxillary robotic-assisted thyroid surgery: technique and results of a preliminary experience on the Da Vinci Xi platform

**DOI:** 10.1186/s12893-019-0473-0

**Published:** 2019-04-24

**Authors:** Francesco Paolo Prete, Rinaldo Marzaioli, Serafina Lattarulo, Daniele Paradies, Graziana Barile, Maria Vittoria d’Addetta, Giovanni Tomasicchio, Angela Gurrado, Angela Pezzolla

**Affiliations:** 1 Department of Emergency and Organ Transplant - Minimally invasive and Endocrine Surgery Unit, University Medical School of Bari, Bari, Italy; 2 Department of Surgical Oncology – IRCCS Istituto Tumori “Giovanni Paolo II”, Bari, Italy; 3 Department of Biomedical Science and Human Oncology - Emergency, Digestive and Endocrine Surgery Unit, University Medical School of Bari, Bari, Italy

**Keywords:** Robotic-assisted, Trans-axillary approach, Remote access surgery, Thyroidectomy, Da Vinci Xi

## Abstract

**Background:**

Robotic thyroidectomy by transaxillary approach (RATS) is regarded as a feasible and safe alternative procedure in selected patients with benign disease or thyroid cancer of low risk, facilitating thyroidectomy with respect to conventional endoscopic approach and offering improved cosmetic results. The Da Vinci Xi surgical system (Intuitive Surgical, Sunnyvale, CA, USA) presents technical advantages over its previous generations, including overhead docking, more compact robotic arms, extended range of motion, and ability for camera to be docked in any arm. This construct supports dissection in smaller spaces with less arm interference and improved view. We present an initial experience of RATS on DVSS Xi in an academic Centre in Italy.

**Methods:**

We conducted a prospective observational study, involving patients with thyroid disease and treated between April 2016 and January 2018. A modified thyroidectomy retractor (Modena retractor, CEATEC Medizintechnik, Germany) was used to lift a musculocutaneous flap and operate gasless. Instrument placement was recorded for each procedure. Each procedure description was broken down into three phases, creation of working space, machine docking with instrument positioning and endoscopic operating technique. Duration of cases was recorded. Patients selected were young women, BMI < 30, thyroid nodule < 5 cm, cytology TIR2 to TIR4 (TIR4:only nodules < 1 cm diameter).

**Results:**

Twelve RATS were performed within the learning curve for the robotic technique, 10 lobectomies and 2 total thyroidectomies. No patients required reintervention. Mean duration of surgery was 198.9 min for lobectomy and 210 for thyroidectomy. The same surgical team performed all procedures. No patients presented surgery-related complications, mean stay was 3 days. Decrease in operating time was observed after 8 cases along with more precise preparation of working space. Four arms were used in the first 10 procedures then only three. No recurrent laryngeal nerve dysfunction, no seroma or haematoma were recorded. One patient had transient hypocalcaemia after total thyroidectomy.

**Conclusions:**

Since the early phases of a preliminary experience RATS appeared a safe alternative to open thyroidectomy. Uptake of technique was quick on Xi platform with few technical tweaks over techniques described for Si machines. Careful patient selection is crucial.

**Trial registration:**

Retrospectively registered on 20 july 2018 . Trial registration number: researchregistry4272.

The Research Registry: https://www.researchregistry.com/browse-the-registry#home/registrationdetails/5b517f08dbc2045aefd7f9b4/

## Background

Minimally invasive techniques, developed with an aim to reduce postoperative pain, improve on cosmetic outcomes, and potentially reduce the length of hospitalization, were introduced at the beginning of the century to treat small thyroid nodules [1, 2]. Technical innovation, improvements in operating techniques and minimally invasive instruments, and an advanced understanding of the endoscopic anatomy of the neck, helped thyroid procedures move from conventional to transcervical endoscopic-assisted thyroidectomy [1], transaxillary [2],bilateral axillo-breast [3] and retroauricular approaches [4].

The research behind these transitions was fuelled by the aim to limit or avoid a neck scar: while many patients may well tolerate a cervical scar, potential complications of a scar such as paraesthesia/dysesthesias, local pain, hypertrophy of the scar and keloid formation have all been described as adverse outcomes of an open thyroidectomy. The target of current endoscopic procedures is repositioning the scar to a less visible location.

The transaxillary approach features incisions that are concealed in the axilla when an arm is at rest. Before the introduction of surgical robots, the implementation of endoscopic thyroidectomy from a remote access was associated with a number of technical difficulties [5, 6].

Limitations such as lengthy learning curve, availability of endoscopic instruments with limited degrees of freedom, difficult manipulation of delicate structures in a constrained space, flat, bidimensional images or troubles in maintaining a working space [7]. Complications as increased CO_2_ partial pressure, tachycardia and subcutaneous emphysema were also reported in cases of carbon dioxide insufflation [8, 9]. Extra-cervical techniques also typically demand more flap dissection, tissue interruption, and longer operating times.

Many surgeons embraced robotic surgery to overcome the limitations of endoscopic thyroidectomy [10–12]. Robotic systems provide a 3-dimensional magnified view of the surgical field, fine motion scaling, filtration of hand tremor, and accurate and multiarticulated hand-like motions. The technique for robotic-assisted gasless transaxillary thyroidectomy (RATS) [13] pioneered by a South Korean group was devised to be scarless neck surgery,and offered less pain and a faster recovery [14] gaining popularity rapidly [15–19].

The Da Vinci Surgical System (DVSS) model S and Si (Intuitive Surgical, Sunnyvale, CA, USA) have already been integrated into the field of head and neck surgery to facilitate the performance of thyroidectomy using the minimally invasive extra-cervical approaches [8, 10, 12, 20–22].

The fourth generation DVSS Xi (Intuitive Surgical, Sunnyvale, CA, USA), was introduced as an advanced platform offering technical advantages over the earlier version. These include overhead docking, allowing for surgery in a wider field without repositioning the equipment, smaller robotic arms, improved range of motion, and ability to dock the camera in any arm, a concept designed to support dissection in smaller spaces without arm interference and better clarity of view. Surgical imperatives (functional preservation of the recurrent laryngeal nerve [RLN] and parathyroid glands) are the same as in conventional surgery, but with differences in terms of patient positioning, equipment and surgical technique. This study presents an initial experience with RATS on the DVSS Xi platform, based on the first12 consecutive cases performed in an academic Institution in Italy. To the best of our knowledge, there are no other reports of RATS delivered on such platform at the time of writing,

## Methods

We conducted a prospective observational study, involving patients with thyroid disease and treated between April 2016 and January 2018 at Policlinico di Bari University Hospital, Division of Minimally Invasive Surgery, Bari, Italy.

Criteria of inclusion were female sex, thyroidectomy for nodular disease with cytology score ranging from benign (TIR2) to suspicion of differentiated thyroid neoplasm (TIR4). Nodules had to be < 5 cm in diameter (< 1 cm if suspect for differentiated ca), situated in a lobe < 7 cm of maximum diameter. Preoperative diagnosis of all patients with thyroid nodules was established by ultrasound scan and fine-needle aspiration biopsy in accordance with recent American Thyroid Association guidelines [23].

Exclusion criteria included a history of neck surgery, thyroid pathologies such as non-differentiated or locally advanced cancers, Hashimoto’s thyroiditis, Graves’ disease, substernal or retropharyngeal goiter, cervical or distant nodal metastases [19].

As it has been suggested on initial implementation of the robotic approach, patients were screened for contraindications such as limitations of mobility of the neck or shoulder, rotator cuff disturbances, cervical spine disease, previous surgery in the neck, chest wall or axilla, and other potentially complicating conditions like obesity. In particular, further contraindications were: distance between the sternal notch and lateral edge of the pectoralis major muscle > 18 cm and BMI > 30 kg/m^2^ (relative), history of neck and pectoral surgery or irradiation, thyroiditis (absolute) [24].

For each patient demographics (age, sex, BMI, ASA score), nodule size (mm), operative time (min), intraoperative blood loss (cc), duration of hospital stay (days), postoperative complications (n/total number of patients), final pathology results (benign/malignant, size, stage), and cosmetic satisfaction were assessed.

Intraoperative components of the initial procedures, including flap time (preparation of working space), docking time, console operative time, and total operative time, were recorded in minutes.

Total operative time was defined as time from skin incision to completion of skin closure, including docking and undocking of the robot. Docking time was defined as the length of time required to dock the robot armed with instruments. Console operative time was defined as the length of time needed to complete thyroid resection after docking the robot.

Video laryngoscopy was used preoperatively in all patients to assess recurrent laryngeal nerve (RLN) function. In case of signs of RLN dysfunction (hoarseness) laryngoscopy would be repeated until return of RLN function was documented. All values are expressed as mean ± SD, ranges, or absolute numbers.

The DVSS Xi Robot console (Intuitive Surgical, Sunnyvale, CA, USA) was operated by one senior surgeon, experienced in endocrine and minimally invasive surgery [25, 26].

Patients were enrolled using the approved selection criteria after full approval of the institutional review board (“Comitato Etico”) of Policlinico di Bari, School of Medicine. Informed consent for research use of data was obtained from all patients participating to this study. All identification data of the patients were removed from medical records prior to data collection. De-identified data were used, and it was not possible to trace any of the data to the actual individual. Only information required for coherent description of cases was extracted. Data in electronic format were accessible to authorized personnel only. No intervention other than recording, counting and analysing of data took place. Consent to publish was obtained by participants, to report individual patient images relative to patient positioning and postoperative outcomes.

## Operating technique

### Room setup (Figs. 1 and 2 a,b,c)

**Fig. 1:**
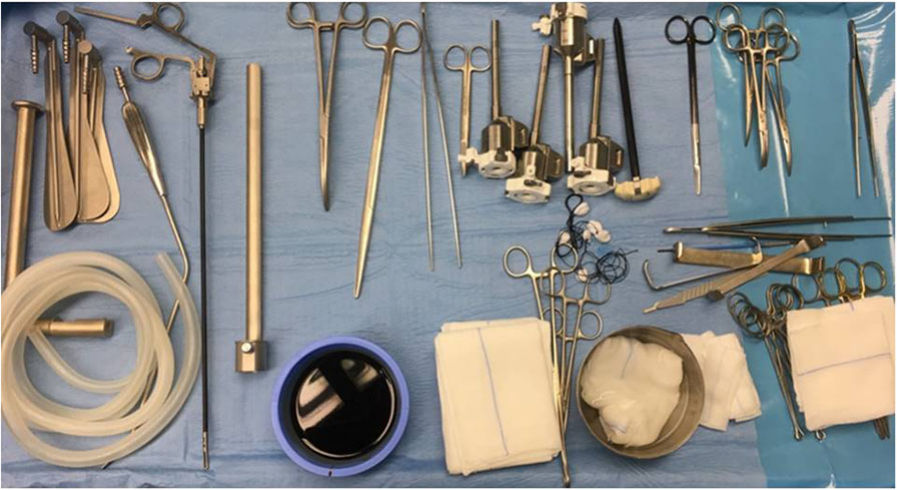
Instrumentation set for preparation of working space (scalpel with blade n. 15, long Klemmer tissue forceps, short and Long DeBakey forceps, short and Long Metzenbaum scissors, Farabeuf and Langenbeck wound retractors, Modified thyroidectomy retractor (Modena retractor, CEATEC Medizintechnik, Germany), 30° Endoscopic camera, 5 mm Johann and bipolar forceps, monopolar electrocautery with long tip extension, vessel sealing device, endoscopic suction/irrigation device)

**Fig. 2:**
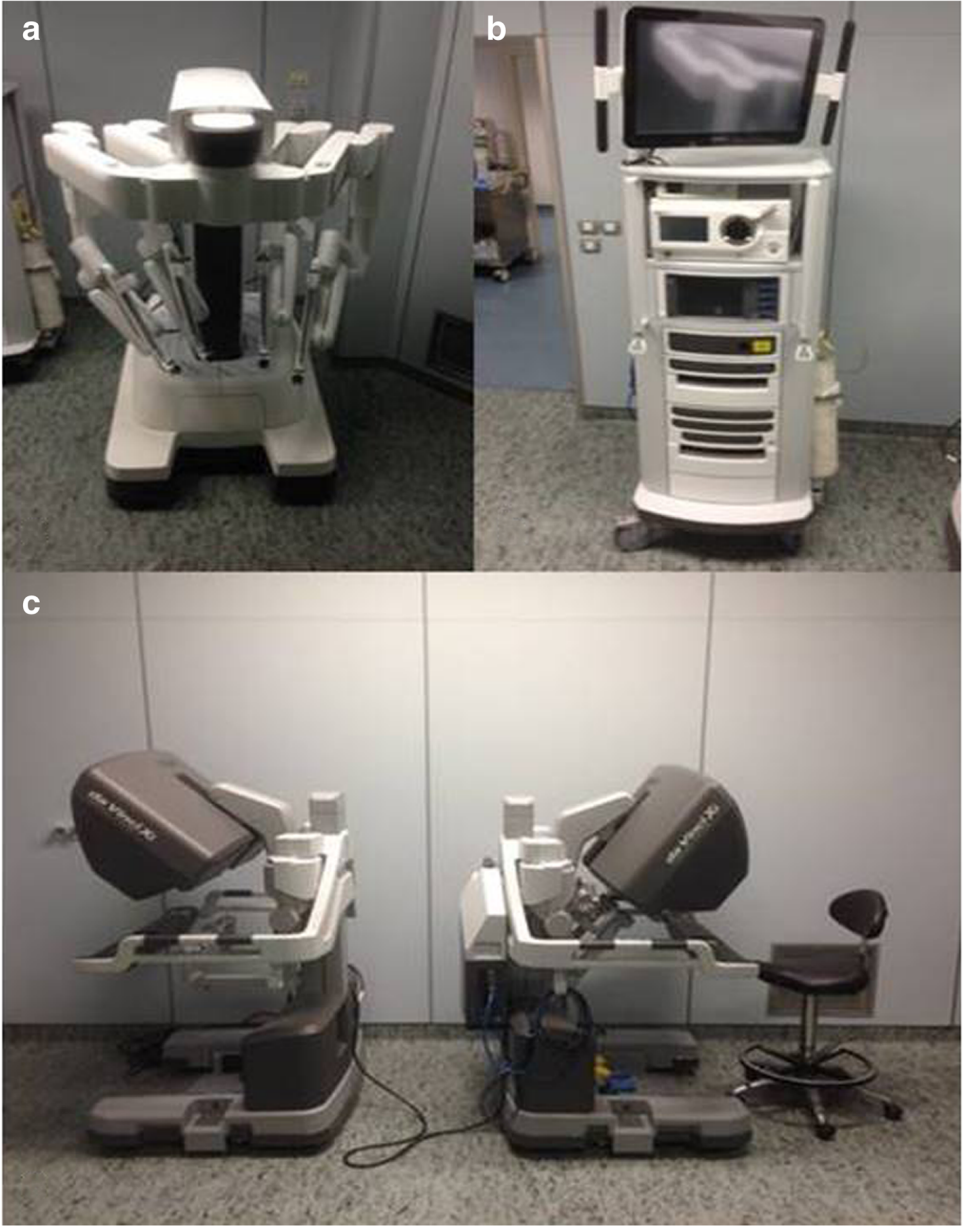
Da Vinci Xi Surgical System (Intuitive Surgical Inc., Sunnyvale, CA, USA): **a** Patient cart; **b** Da Vinci Surgical System Xi: Video cart; **c** Da Vinci Surgical System Xi: Surgeon console

The anesthetist controls the patient’s head and airways from the head of the table. Equipment has been checked prior to patient’s entry (Figs. 1 and 2). At the beginning of procedure, the patient cart of the robotic surgical system, sterile draped, is distant from the operating table (depending on the side where the surgical incision is being performed, an assistant on the contralateral side will help to create the working space and position the retractor); the patient cart will later be docked on the side of the operating table opposite to the incision. The assistant and the scrub nurse will be on the ipsilateral side to the incision with the instrument table. The video cart or an additional video monitor should be placed in clear view of the assistant. The surgeon’s console is typically located sideways in the OR.

### Patient positioning (Fig. 3 a,b)

The patient is positioned supine under general anesthesia. Following intubation, slight hyperextension of the neck is obtained using a shoulder roll underneath the patient’s shoulders. The arm contralateral to the incision site is padded and rests adjacent to the patient’s body. The arm on the same side of the incision is placed in an arm board extended above the head to expose the axilla, without exceeding 125° of antepulsion, with the elbow flexed to 90°, and with proper padding.

**Fig. 3:**
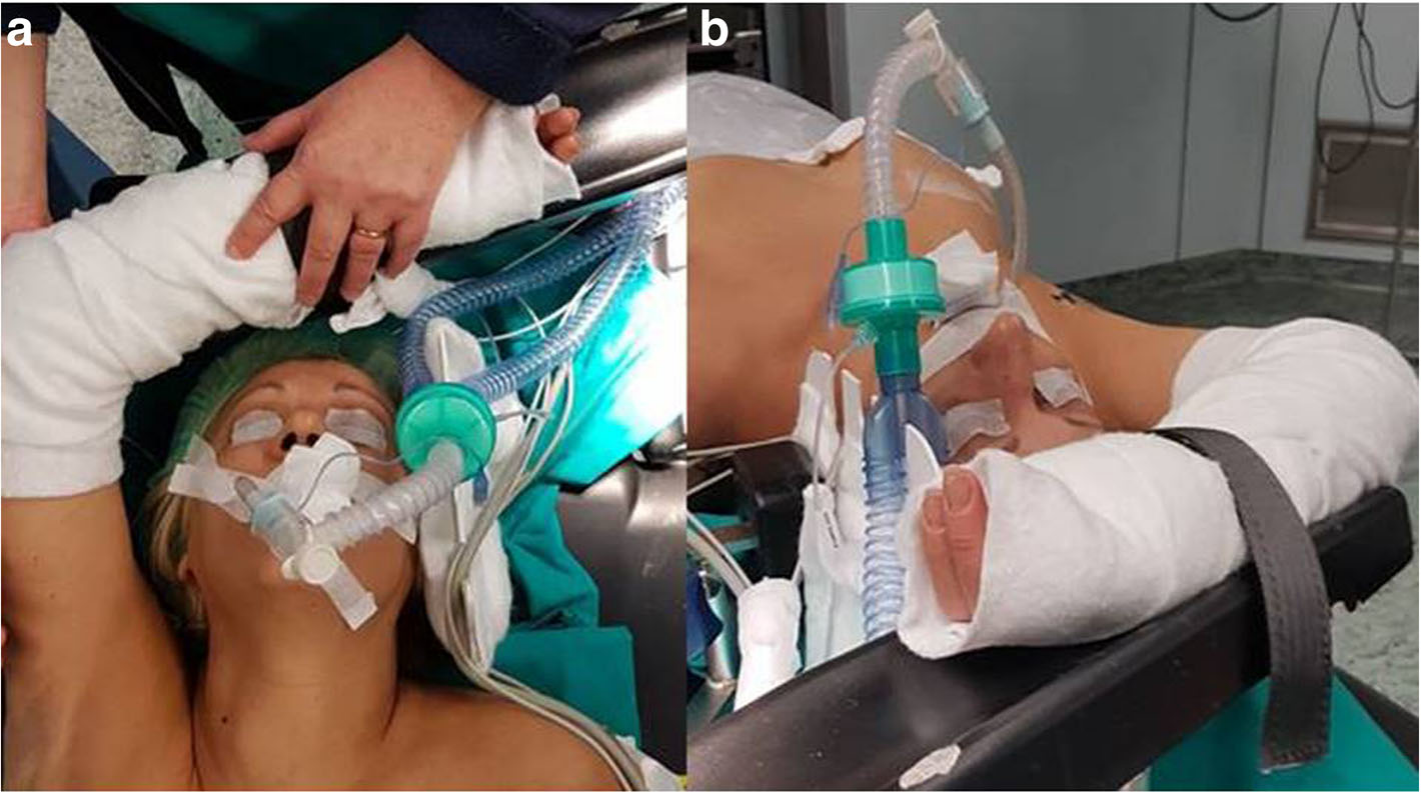
**a** Patient positioning: the ipsilateral arm, padded, extended cephalad and flexed over the head is placed on a dedicated arm rest; **b** Patient positioning: extended ipsilateral arm secured in position

Before general anesthesia, position of the arm is checked in the operative room to prevent neurapraxia of the brachial plexus. Adequate padding is also placed around bony prominences on the arm, under the neck, shoulder, and between the raised arm and neck.

Under this setup the axilla gains adequate exposure, and the distance between the axillary skin and the thyroid gland is shortened. Good positioning is essential for exposure in this procedure, so patients with limited range of shoulder or cervical mobility require careful positioning and, in severe cases, may not be suitable for this procedure.

### Landmarks (Fig. 4 a,b)

The thyroid is identified by palpation and two dots or short lines are designed to mark the sternal notch and the hyoid in the midline, respectively. An imaginary line connecting these two points demarcates the medial boundary of dissection. Two other imaginary lines guide the positioning of the surgical incision: the first runs transverse from the sternal notch to the ipsilateral axilla, immediately lateral to the pectoralis major, denoting the inferior limit of the surgical incision; the second line is oblique and runs from the hyoid marking to the axilla, bearing an angle of 60° from midline, and marks the upper limit of the incision. These same lines define the limits of the dissection space. A 5- to 6-cm line is then marked in the axilla at the lateral border of the pectoralis major muscle. The arm is placed into its natural position to confirm that the future incision will hide in the axilla postoperatively, then it is secured in place. The site for an additional, single trocar incision, is marked down the line of the future axillary incision. Sterile drapes include the axillary, anterior cervical and prepectoral regions, allowing surveillance of skin integrity during the incision and possible conversion to anterior neck surgery.

**Fig. 4:**
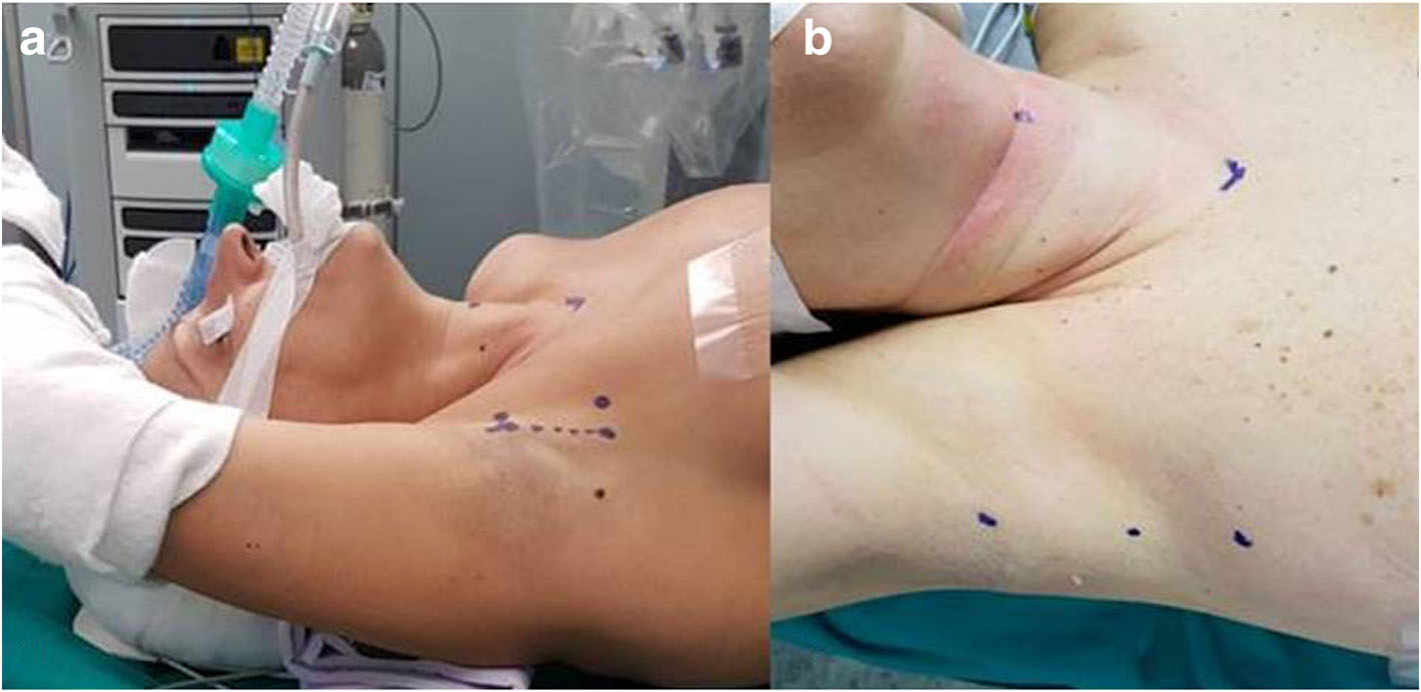
**a** Landmarks: two dots mark the sternal notch and the cricoid cartilage, the mark at the inferior end of the future axillary incision is connected to the sternal dot by an imaginary transverse line; the mark at the upper end of the future axillary incision is connected to the cricoid dot by an imaginary line forming an angle of approximately 60° with the neck midline; **b** Landmarks: detail of the markings for the axillary incision (cephalad and middle dot) and for an accessory trocar (caudal dot) for retraction

#### Surgical technique

The surgical procedure included creation of a working space by flap formation and suspension -under direct and then endoscopic vision-, robot system docking, and console work.

### Creation of the working space(flap creation)(Fig. 5 a,b,c,d)

An incision 5–6 cm long is performed along the line marked in the axilla. The flap is created in a plane superficial to the pectoralis fascia. Dissection is performed initially under direct view with electrocautery, above the pectoralis major, using retractors consecutively longer to elevate the skin and the subcutaneous tissue. A dedicated thyroid retractor, a modified thyroidectomy retractor (Modena retractor - MR, CEATEC Medizintechnik, Germany), is mounted on bed opposite the incision side and deployed under the flap, to lift the flap and maintain the working space. The retractor blades are progressively changed adapting to deeper dissection. Dissection, continuing now under endoscopic camera assistance, proceeds until the clavicle is identified and then continues medially down to the sternal notch, by using a combination of laparoscopic instruments (hook diathermy, bipolar forceps and/or harmonic scalpel, suction cannula) and a small sponge mounted on long Klemmer forceps for tissue retraction. Next, dissection along the lower aspect of the platysma continues until the sternocleidomastoid (SCM) muscle is identified. At the end of the tunnel the two ends of the sternocleidomastoid muscle are retracted, the strap muscles are lifted up with the MR and the ipsilateral jugular vein is exposed along with the thyroid. It is important to carefully avoid an internal jugular vein injury during this part of the dissection, while afferent vessels are sealed. Once the thyroid lobe and lateral edge of strap muscles are visible on the side of the incision, electrocautery or vessel sealing device are used to dissect the strap muscles off the thyroid. The retractor lift is aimed at providing an adequate working space with full visualization of the thyroid; the height of such lift should be at least 4 cm at the opening. The anesthesiologist should ensure that the patient has adequate padding around the neck and shoulders after the retractor is secured.

**Fig. 5:**
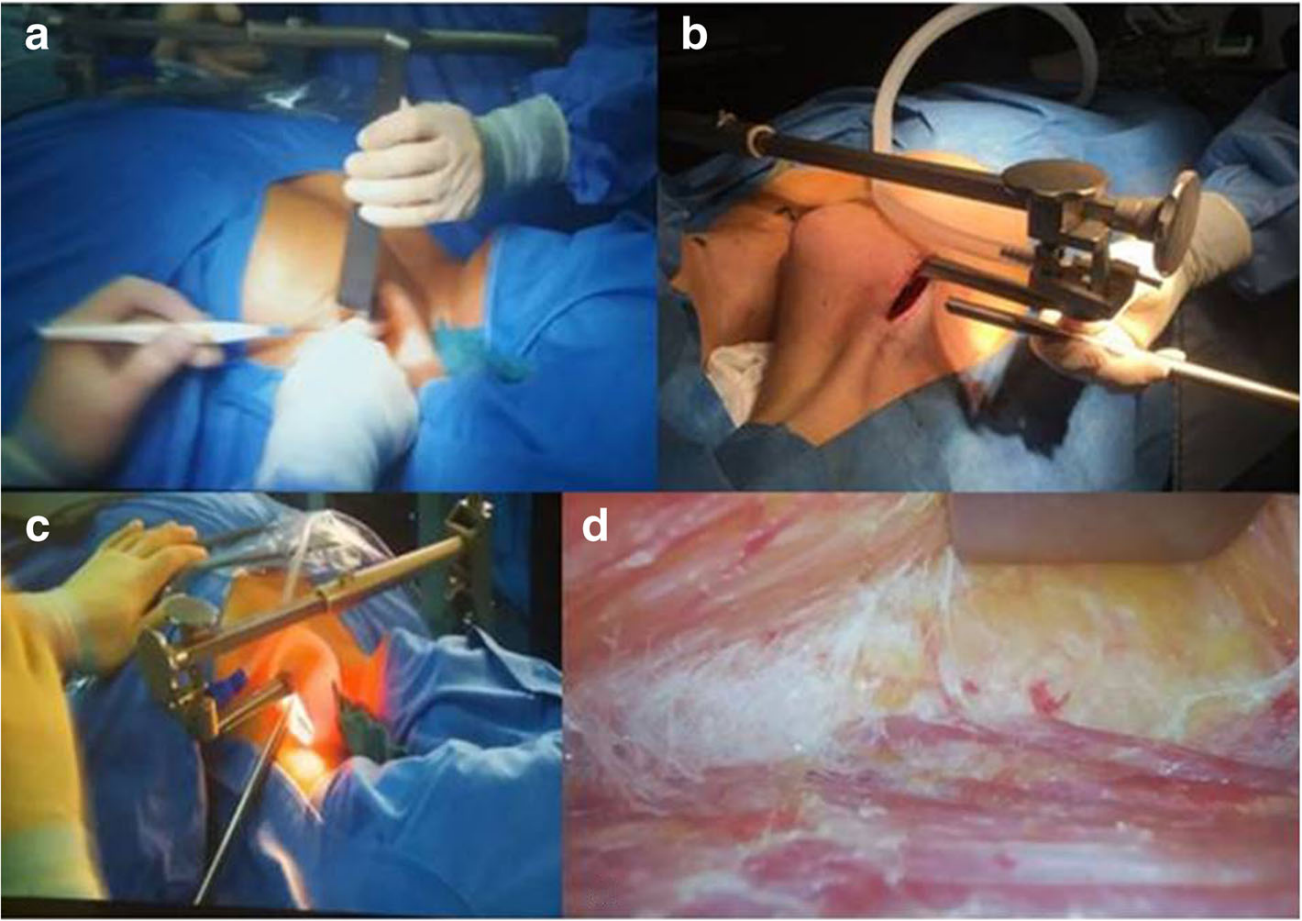
**a** Creation of working space: dissection of the subcutaneous tissue under direct view; **b** Creation of working space: moving deeper with stable retraction. The Modena retractor in place with suction tube mounted, preparing for dissection under endoscopic camera assistance; **c** Creation of working space: dissection under camera assistance can help limit the extension of the axillary incision; **d** Creation of working space: endoscopic view of dissection along the greater pectoralis fascia

### Robot docking(Fig. 6)

The Da Vinci® Xi surgical robot (Intuitive Surgical Inc.) consists of a surgeon console that controls the instrument holder on the patient side and a high definition telemonitoring screen. The four arms of the robot carry: 30°degrees endoscope, Maryland dissecting forceps, Prograsp fenestrated forceps, Harmonic ACE shears insert (Johnson & Johnson Medical, Belgium, EU).The sterile draped robot is docked to the side opposite of the lesion or incision. Due to the degrees of freedom (elevation, rotation) of the DVSS Xi boom from which arms are deployed, no aligning is required between robot cart and incision/MR, and the fulcrum of the rotating boom is centered to the middle of the axillary incision (in DVSS Si the whole robotic cart needs aligning and centering). The robotic arms are deployed and three robotic trocars are positioned through the axillary incision with the camera being placed first. A 30° stereoscopic endoscope is docked first in the middle of the incision (DVSS Xi arm n.2), with the camera directed upwards with respect to the floor (20° to 30°) and the patient’s feet (10° to 20°) (camera should be positioned so that its robotic arm-end or bottom end outside the wound should be low, while its tip inside the wound should be high with respect to the plane od the operating bed), in the center of the lower border of the incision, thus allowing maximum range of arms motion and avoiding collision.

**Fig. 6:**
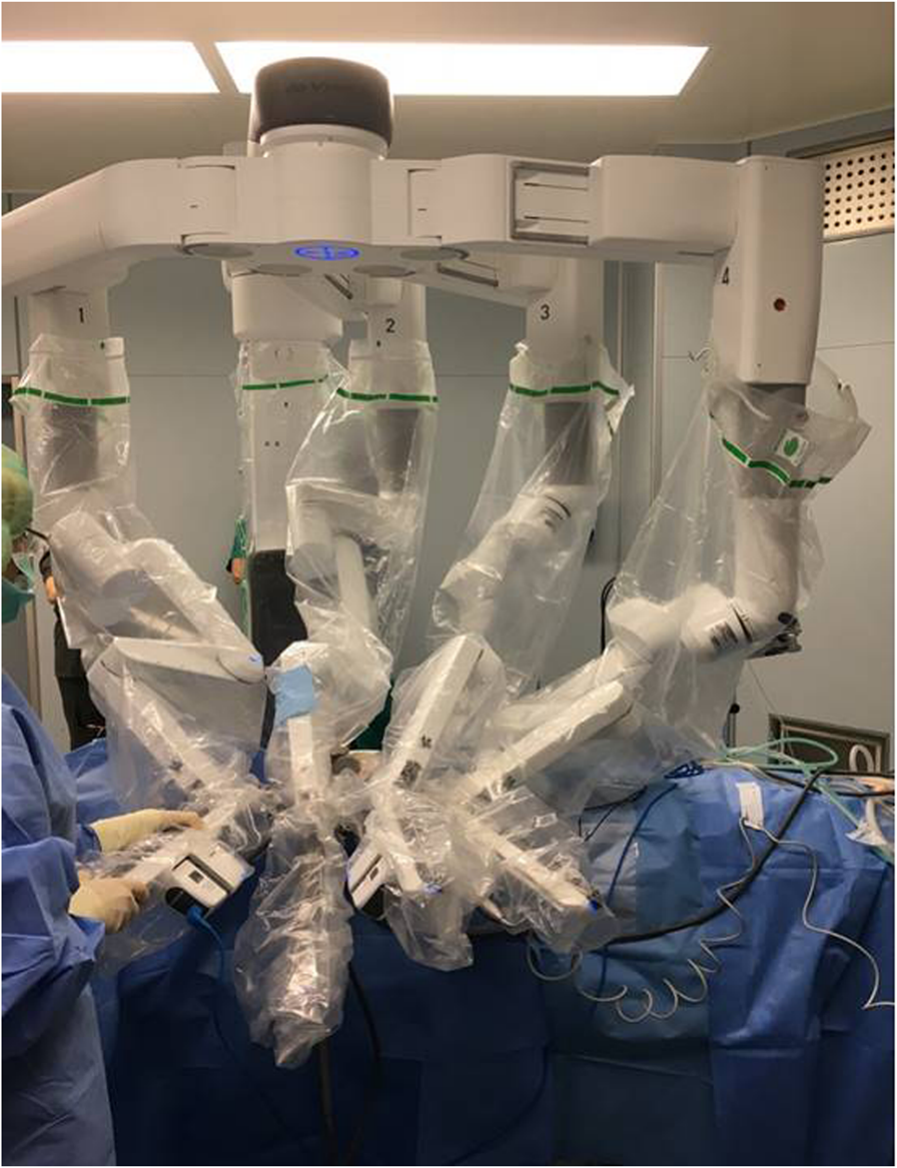
Docking: Da Vinci Xi docked in position with four arms engaged

Then an 8-mm curved Harmonic shears and a Maryland forceps are deployed on either side of the camera (as opposed to 5 mm instruments in DVSS Si) and directed towards the floor ((DVSS Xi arm n.1 and 3), the Harmonic shears in a position that should match the dominant hand of the surgeon. The angles with which these instruments are placed are essential to prevent instrument conflict within the wound: the two instruments and the camera should form an equilateral pyramid with a summit situated in the working space. The centre of the instrument area, the MR, thyroid and camera should all be situated in the same plane.

A fourth instrument, a Prograsper, is positioned through a small incision a little caudal to the lower end of the axillary incision and used for retraction when 4 trocars are used. If three trocars are used, a Prograsper is switched with the Maryland forceps in its standard position.

### Console time(Fig. 7)

The surgeon operates the console while the assistant and scrub nurse are placed next to the patient to ensure the absence of any conflict of the robotic arms. The assistant helps retracting tissues, aspirates smoke, irrigates and introduces instruments into the operative field. The Maryland forceps and harmonic shears are used for dissection (harmonic is also used to coagulate and section blood vessels), while the Prograsp fenestrated forceps is used to retract the lobe of the thyroid.

**Fig. 7:**
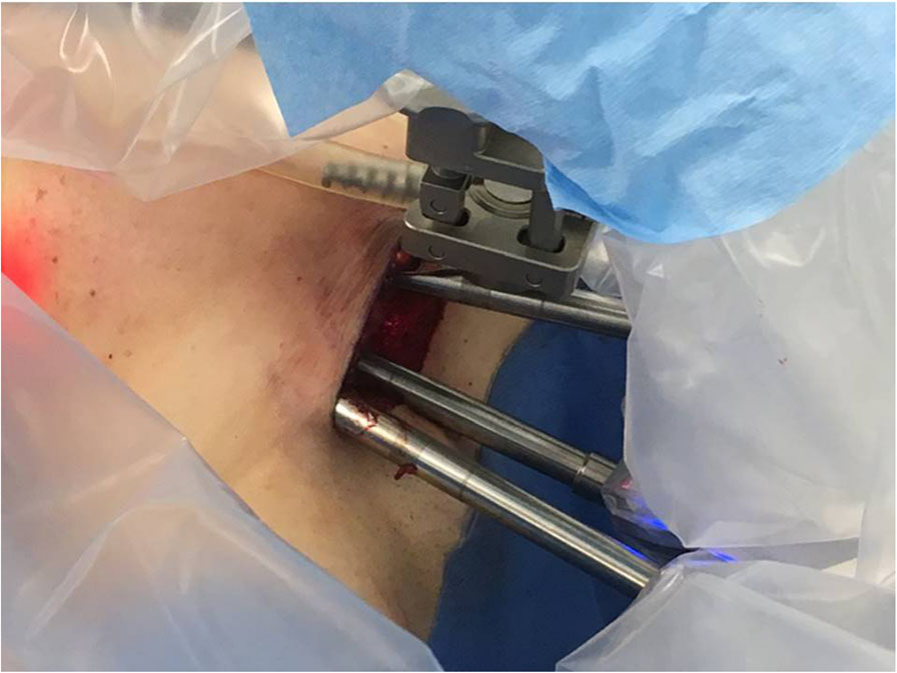
Detail of dissection using three robotic arms through the axillary incision (camera and instruments are ideally located at the three sides of an equilateral pyramid)

The upper pole is retracted medially and inferiorly with the Prograsper. Then the superior vessels of the lobe are dissected using the Maryland forceps, and divided with the Harmonic close to the gland, to prevent injury to the superior laryngeal nerve (external branch). After freeing the upper pole from the cricothyroid muscle, the superior parathyroid gland is identified and preserved. Then the thyroid is retracted medially by repositioning the Prograsper, and the middle thyroid vein, once dissected, is divided with the Harmonic shears. The recurrent laryngeal nerve (RLN) is identified at the tracheo-esophageal groove and demonstrated throughout its course up until insertion into the crico-thyroid muscle, always considering the possibility of a non-RLN anatomical variant when dissecting the tracheo-oesophageal groove on the right hand side. Dissection proceeds then to the inferior pedicle, which is isolated using the Maryland forceps; the inferior parathyroid gland is identified and preserved, and then the inferior pedicle is divided using Harmonic shears. Then the thyroid lobe is lifted and carefully dissected medial to the course of the RLN and finally off the trachea until the contralateral side is reached. The lobo-isthmusectomy is completed by dividing the thyroid between the isthmus and the contralateral lobe with Harmonic, and specimen is removed through the incision. Haemostasis is checked with a Valsalva manoeuvre.

For total thyroidectomy, after extracting the ipsilateral lobe and isthmus, dissection continues freeing the contralateral lobe from the trachea until the contralateral tracheoesophageal groove is reached. After the RLN is identified, the superior and inferior thyroid pedicles are dissected; then RLN is followed until its insertion into the cricothyroid membrane; the rest of the thyroid lobe is dissected and removed via the axillary incision.

At the end of the procedure a Jackson-Pratt drain is positioned in the thyroid bed, running through an incision next to the axillary. Subcutaneous interrupted stitches and continuous subcuticular suture close the axillary incision in two layers.

## Results

In this study 12 patients underwent transaxillary robotic-assisted thyroidectomy. All patients were females with a mean age of 44.9 years (age range, 31–63 years). Ten procedures were lobectomies, and two were total thyroidectomies. Indication to surgery was in ten cases a nodule scoring TIR3 (indeterminate) on preoperative cytology, TIR4 in one case, and one nodular goitre. The mean nodule size was 23.6(range 9–40) mm. Preoperative laryngoscopy was unremarkable in all patients.

The mean total operative time was 190.5 min (range 75–377), the mean docking time was 23.6 min (range 8–37), and the mean console time was 78.3 min (range 27–175). The mean time for creation of the working space was 88.6 min (40–155) and, together with all the other time figures, decreased significantly after the 8th procedure. There were no conversions to open surgery. The first three cases, including two total thyroidectomies, were proctored by an endocrine surgery team with senior expertise in RATS. Four robotic ports were used for the first 10 consecutive cases, including both TT, while three arms were used to complete the last two lobo-isthmectomies.

**Fig. 8:**
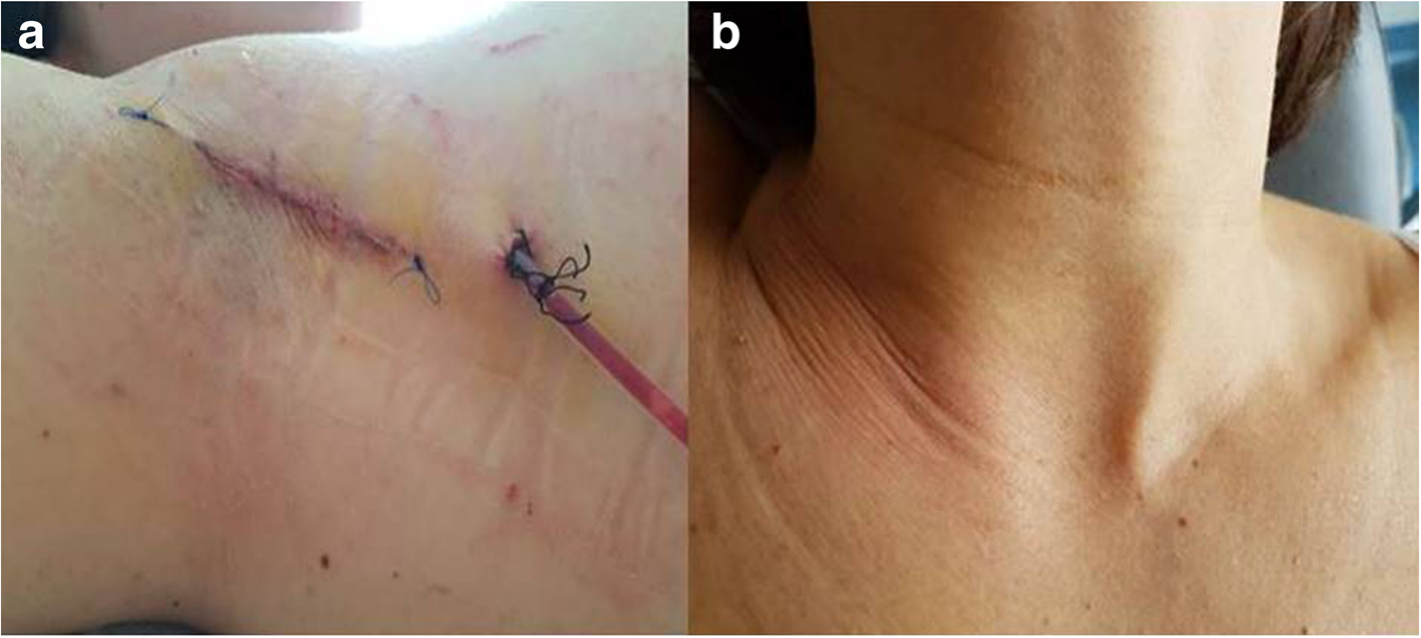
**a** End of procedure. Detail of the sutured 5 cm incision, concealed in the axilla; **b** Cosmetic outcome at the level of the neck on 1st postoperative day

The mean blood loss was 25 (range 10–100) mL. One of the 2 patients who underwent total thyroidectomy (8.3% of all procedures) was found to have hypocalcemia. Her calcium was found to be at a level of 6.9 mg/dL the day after the operation (reference range, 8–10.1 mg/dL), although she was asymptomatic. She was discharged with a calcaemia of 8.2 mg/dl and was normocalcemic 2 weeks postoperatively after supplementation of oral calcium at home. Two patients experienced mild discomfort at the level of the incision that resolved spontaneously after 24 h. There were no other perioperative or postoperative complications. All patients tolerated the procedure well and could be discharged home in 2 to 3 days (mean 2.25). (Fig. 8 a,b).

There was no evidence of recurrent laryngeal nerve dysfunction, either during hospitalization or at follow-up. At follow-up, because of the concealed incision site, cosmetic results were considered subjectively excellent in all cases.

Histological examination revealed 5 cases of papillary microcarcinoma, all pT1a and < 6 mm diameter, of which only one was fully anticipated by TIR4 cytology. None of the lobectomies resulting in such diagnosis required secondary contralateral transaxillary total thyroidectomy. No lymph node dissection was performed in the absence of any clinical or ultrasound evidence of lymphadenopathy (cN0).

Analgesic consumption was not different from that observed after conventional surgery. No postoperative hematomas or seromas were observed during the 30 days follow-up.

## Discussion

The robot-assisted thyroid surgery was found in several studies to be as safe and oncologically sound as in a classical open approach [27, 28]. Compared to the open or endoscopic approaches, the robot-assisted transaxillary approach has a number of technical advantages: the robotic system offers three-dimensional vision with up to 10 times magnification of the surgical field and wristed instrumentation, supporting a wider range of endoscopic maneuvres; it offers with motion scaling and tremor filtration; improved surgical ergonomics contribute to reduce discomfort to the surgeon, as compared to open and endoscopic surgery; with the robotic system the surgeon can control both the endoscope and operating arms simultaneously through the console.

The transaxillary endoscopic approach to thyroidectomy offers an operative view similar to that of open surgery, empowering identification of the RLN and the parathyroid gland and making it relatively simple [29]; many authors versed in endoscopic neck surgery regarded the improved visualization as a major benefit of the robotic approach [30].

The Xi generation of the da Vinci surgical system provides some additional benefits over the previous version, including narrower robotic arms, improved range of motion, and the possibility to dock the camera in any of the four arms. Such construct aim at supporting dissection in narrow spaces avoiding arm interference. Of note, while the DVSS Si can use 5 mm instruments, the Xi system only accepts 8 mm ones, a potential disadvantage where availability of endoscopic space for dissection is so important; however we did not find this to adversely impact thyroid dissection. We found that RATS can be successfully performed with excellent visualization using the Xi robotic system. The advantages to the thyroidectomy as perceived on exposure and ease of dissection are further accentuated with the overhead docking capabilities, with no further need for equipment repositioning.

Robot-assisted transaxillary approach has been found to be conducive of better patient outcomes, including cosmetic satisfaction with a reported reduction in postoperative pain [13, 21, 31–33], post-thyroidectomy voice change [34], and swallowing discomfort [7, 31]. By avoiding a visible neck scar the transaxillary approach offers an immediately perceptible cosmetic advantage over conventional thyroidectomy. Scar satisfaction appears not related to the appearance of the resulting scar but to the distance of the scar from the neck, which makes this an attractive approach to young female patients, in particular to those with a propensity toward formation of a keloid scar.

Compared with a two-incisions technique, the robot-assisted approach with a single axillary incision by elimination of anterior chest wall incision has been found to be technically safe and feasible [13]. When a single axillary incision is sufficient to prevent instrument conflict and a second subareolar or parasternal incision is avoided, cosmetic results are improved too, the procedure is rendered less invasive and there is limited interference with subsequent mammographies or surgery in this region [13]. When two incisions are necessary, the use of an in-line instruments access from axilla incisions is also aimed at designing prepectoral dysaesthesia out of the range of potential complications. The variables that limit instrument conflicts are a clear definition of the incision, creation of working space, and positioning of the robot, together with intraoperative surveillance by the assistant [17, 19] In our experience five-six cases were needed to begin controlling such variables.

The robotic approach portrays a longer operative time as compared to a conventional open approach. However, studies have shown that operating time as a variable depends in turn on the surgeon’s experience and on the frequency with which robotic thyroidectomy is performed [27, 35]. There is evidence for gradually decreasing operating times, with a plateau after 20 cases of robotic less-than-total thyroidectomies, when these are performed by surgeons with little or no experience in endoscopic surgery [36]. Within the scope of this limited experience, well within the perceived learning curve (20–30 cases), improvement was felt to be achieved in two elements of the timing of the surgical procedure, the robot docking time and the console time.

The minimal incision is 6 cm [37] and dissection must ascend sufficiently high to control the superior thyroid pedicle. Piccoli et al. described a shorter incision when endoscopic vision is used during the creation of the working space together with the Modena retractor, as such retractor can be used since the start of the procedure and can be handled by just one surgeon, a model that we elected to follow [38].

RATS has been shown to introduce new potential complications, as chest paresthesia (for extensive skin flap dissection) [39] or a risk of brachial plexus neuropathy owing to the position of the lifted arm: placing the arm in a flexed overhead position and avoiding overextension of the shoulder may reduce the risk of stretching the nerves. Intra-operative nerve monitoring may further reduce the chance of brachial plexus injury, by identifying impending damage to of the ulnar, radial, and median nerves and enabling patient repositioning [27].

A distance of 4 cm between the anterior and posterior limits of the incision, and a space ≥1 cm between the anterior aspect of the thyroid and the retractor have been recommended [39] .

We undertook robotic –assisted thyroid procedures in carefully selected patients, mainly presenting with indication to surgery for an indeterminate thyroid nodule [40]. Nodules were defined preoperatively by fine-needle aspiration citology, with the known margin of uncertainty related to the procedure [41] . Nodule volume superior to 5 cm was contraindicated, to ease handling and visualization of structures in the context of the anatomy as presented during the first cases of the robotic approach. However with an ever increasing experience, relative contraindications are no longer regarded as such by proficient surgeons [42]: expert Groups routinely perform RATS on patients with Hashimoto’s thyroiditis and Graves’ disease or breast implants [43]. RATS has also been considered to be equally safe irrespective of the presence of BMI > 30 [44], although morbidly obese patients may pose significant challenges regarding positioning, dissection, and placement of the retractor needed to keep the working space open .

Conversion from robotic to open surgery has been noted to occur in very large plunging goitres [43].The mean size of the nodule in our resected specimens ranged from 10 to 40 mm. Within these boundaries, all surgical procedures were successfully completed without need of converting to the open approach. We could view the RLN and the parathyroid glands clearly, and we found no evidence of damage to either of these structures in any of the performed procedures. One of the two patients who had total thyroidectomy developed transient postoperative hypocalcemia, with no evidence of resected parathyroid tissue on pathology specimen. Postoperative hypocalcemia has been associated to longer operating time than 120 min in female patients, owing to possibly increasing risk for vascular supply to the parathyroid glands with prolonged dissection [45]. The mean operative blood loss in this series also appeared lesser than that reported in endoscopic [46–48] or open thyroidectomies [49, 50]. In large series, paralysis of the recurrent laryngeal nerve has been reported in 0.3–0.8% of cases, while transient recurrent laryngeal nerve paralysis in 2.2% of cases [8, 13]. No evidence of RLN dysfunction suggesting either permanent or transient paralysis was observed in our series, with no visible trauma to the recurrent laryngeal nerve and despite nerve monitoring which has been considered essential [51]. Although certainly satisfactory, at this stage of our experience we consider such outcome as an attribute of a very small series of cases and careful case selection.

Compared to open procedures, RATS requires a longer operating time, which decreases with experience [14]; our long operating times, other than depending solely on developing operator experience, was also impacted by the slow transition between different steps of RATS typical of surgical, anaesthetic and nursing teams that are still coagulating around a newly introduced procedure. As in our case surgeons could benefit from, and hospitals may require, having an experienced robotic surgeon proctor cases, particularly if the operating surgeon has not previously used the robot in other settings. Novel and updated robotic platforms as the DVSS Xi, controlled by high-volume surgeons operating as part of a multidisciplinary robotic team in specialised centres, may help on one side to expand use and offer of robotic thyroidectomy by trans-axillary approach, while supporting new, progressively less invasive routes to thyroidectomy on the other, up to a truly scarless procedure, as in the transoral approach described so far [52].

## Conclusion

Robot-assisted transaxillary thyroidectomy avoids a visible neck scar and decreases postoperative discomfort, swallowing difficulties and neck skin sensory loss. The technique appears to be feasible under satisfactory conditions of safety for carefully selected patients who request this type of surgery to avoid a neck scar, especially when they have a history of keloid scars.

The essential challenge raised by this technique is to allow surgery without excessive conflicts or limitations of movements. When these conditions are met, RATS allows lobectomy similar to that performed via conventional open thyroid surgery. The use of the da Vinci Xi platform provides technological advantages over previous generations improving the ease of the RATS technique. Adequate patient selection, careful port positioning and meticulous dissection are critical. Competency can be obtained within a relatively short period, with low complication rates. We recommend a team approach with more than one experienced surgeon participating during the learning phase and a dedicated OR team for to successfully implement this technique.

Larger, prospective, ethically approved comparative trials between different robotic platforms are necessary to evaluate the benefit-risk balance and excess cost related to this innovative technique.

## Abbreviations

DVSS: Da Vinci Surgical System
MR: Modena Retractor
RATS: Robotic-Assisted Thyroid Surgery
RLN: Recurrent Laryngeal Nerve
SCM: Sterno-Cleido Mastoid (muscle)

## Authors’ information

-Francesco Paolo Prete is consultant general and endocrine surgeon at the Department of Emergency and Organ Transplant - Minimally invasive and Endocrine Surgery Unit, University Medical School of Bari, Italy.

-Rinaldo Marzaioli is associate professor of surgery at the Department of Emergency and Organ Transplant, University Medical School of Bari, Italy.

-Sara Lattarulo is associate specialist general surgeon at the Department of Emergency and Organ Transplant - Minimally invasive and Endocrine Surgery Unit, University Medical School of Bari, Italy.

-Daniele Paradies is general surgeon in training at the University Medical School of Bari, Italy.

-Graziana Barile is general surgeon in training at the University Medical School of Bari, Italy.

-Maria Vittoria d’Addetta is foundation doctor at the Minimally invasive and Endocrine Surgery Unit, University Medical School of Bari, Italy.

-Giovanni Tomasicchio is foundation doctor at the University Medical School of Bari, Italy.

-Angela Gurrado is consultant general and endocrine surgeon at the Department of Biomedical Science and Human Oncology - Emergency, Digestive and Endocrine Surgery Unit, University Medical School of Bari, Italy.

-Angela Pezzolla is associate professor of surgery and head of the Minimally invasive and Endocrine Surgery Unit, Department of Emergency and Organ Transplant, University Medical School of Bari, Italy.
